# Australian Surgery

**Published:** 1831

**Authors:** 


### AUSTRALIAN SURGERY.

### I. ON DISLOCATION OF THE HIP-JOINT. By Mr. BLAND, Surgeon, New South Wales.

MR. BLAND'S attention was drawn to the subject by a case of dislocation of the femur backwards and upwards, when he adopted the following instead of the usual procedure.

The patient being placed on a long low table, on his side, and otherwise in the position customary on these occasions—and the means of counter-extension being prepared in the usual manner; the operator, standing behind the patient, and placing his right thigh firmly between the thighs of the patient, close to the perineum (the dislocation being of the right side) was enabled, by depressing the knee of the dislocated limb, to raise the head of the bone, by which means, aided by a moderate degree of extension, the reduction of the limb was effected with the greatest possible facility.

The prominent advantages which Mr. Bland attaches to the foregoing plan, are, that the hands of the surgeon are at perfect liberty—the one to be used, as required, in depressing the knee—the other, in ascertaining the exact position of the acetabulum, and directing the head of the femur into its place, while the surgeon, unfatigued and unembarrassed, can give his directions, and act with coolness and precision, ensuring the best chance of success.

The extension and counter-extension were made in *this* case (which was recent) by means of mere manual assistance; and an individual was stationed to support the foot of the patient, so as to allow it freely and readily to follow the motions of the knee.

This procedure Mr. B. had followed in every case that occurred to him for some years, and one of the dislocations was of twelve days standing before the operation for reduction. In all these cases he was entirely successful. But at length he was called to a case where the head of the bone had been dislocated into the thyroid foramen for eight days, and in which (from the remote situation where the accident happened) no attempt at reduction had been made. In this case, the above-mentioned mode of proceeding was tried, but failed, and Mr. Bland having also failed by the usual modes of reduction, was obliged to invent a machine for the purpose—a lithographic plan of which he has published in a contemporary journal.

II. LIGATURE OF THE SUBCLAVIAN ARTERY, IN SYDNEY, NEW SOUTH WALES. By MR. BLAND.

It is not a little honorable to Australian surgery to be able to boast of a successful cure of axillary aneurism by so formidable an operation as ligature of the subclavian artery. We can do little more than merely allude to the case in this place. The patient was 63 years of age, a pauper in the Sydney asylum, who first perceived the aneurismal tumour a few months previous to the operation. It was shewn to Mr. Bland and Dr. Fattorini, in December, 1830, when it presented the section of an oblate spheroid, the size of a large orange, extending from the clavicle into the right axilla, attended with considerable and unremitting pain, almost entirely preventing sleep. The man attributed the complaint to a hurt received some time previously. On careful examination, no disease of the heart, or of any other vessel could be detected; and the case having been considered in consultation with Mr. Forster, Dr. Fattorini, Dr. Rutherford, and others, it was determined to tie the subclavian artery, which was done in presence of the above gentlemen. The steps of the operation are not detailed in the paper which we have seen, but only the result. The operation was performed on the 17th Dec. 1830, and the last ligature came away on the 29th January of the present year, leaving the wound entirely healed. Very active depletion was used after the operation. Mr. Bland has omitted to state the appearances of the aneurismal tumour at the time the account closed, which was on the 20th February. We congratulate the Australian profession on this bold and extremely difficult operation being performed at all—but especially as it terminated with success, as far as the operation itself was concerned.

